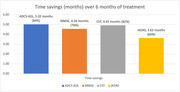# OVERTURE Phase 2 Study time‐savings analysis using composite measures of function and cognition

**DOI:** 10.1002/alz.094669

**Published:** 2025-01-09

**Authors:** Ralph Kern, Benjamin A Haaland, Jessie Nicodemus‐Johnson, Samuel P. Dickson, ZD Kuang, Celine Houser, Mihaly Hajos, Brent Vaughan, Suzanne B. Hendrix

**Affiliations:** ^1^ Cognito Therapeutics, Cambridge, MA USA; ^2^ Pentara Corporation, Salt Lake City, UT USA

## Abstract

**Background:**

The OVERTURE I (NCT03556280) randomized controlled trial (RCT) evaluated Cognito’s non‐invasive device (Spectris^TM^) in mild‐moderate Alzheimer’s disease (AD). Composite measures, such as combined statistical tests (CSTs) and iADRS, combine the joint evolution of function and cognition over time in the population of interest when calculating the combined treatment effect. We estimated time savings of active treatment vs. sham treatment using prespecified ADCS‐ADL and MMSE outcomes, as well as post‐hoc iADRS and CST outcomes using data obtained from the RCT.

**Method:**

The OVERTURE I phase 2 clinical trial (NCT03555280) compared one‐hour, daily, at‐home, active vs. sham treatment for six months (2:1 randomization). We evaluated the ADCS‐ADL and MMSE based on a bivariate linear model for 6‐month change from baseline with correlation structure accommodating a correlation between individual patient change from baseline measures for ADCS‐ADL and MMSE. iADRS was analyzed as described (Wessels et al 2015). The CST is computed based on averages of least squares means for ADL and MMSE as well as treatment effects on each endpoint.

**Result:**

135 participants were screened, 74 were randomized and 53 completed (20 sham, 33 active) the RCT. Safety, tolerability and adherence was confirmed in the RCT, and no serious treatment‐related adverse events or ARIA were observed. Active vs. sham treatment resulted in significant reductions in disease progression over 6 months of treatment (mean difference, 95% CI) in ADCS‐ADL 8.31, 2.85‐13.77; MMSE 2.21, 0.39‐4.04; CST 5.26, 2.17‐8.36; and iADRS 6.65, 0.41‐12.89. Corresponding time savings (months, % time savings) in ADCS‐ADL (5.02, 84%); MMSE (4.56, 76%); CST (4.92, 82%); and iADRS (3.63, 60%) were observed.

**Conclusion:**

The OVERTURE I RCT demonstrated that non‐invasive at‐home (Spectris^TM^) treatment meaningfully reduced the decline in ADCS‐ADL, MMSE, CST and iADRS over 6 months compared to sham treatment and was associated with 60‐84% time savings. These outcomes will be further evaluated in the ongoing HOPE pivotal trial in mild‐moderate AD (NCT05637801).